# Men’s attitude towards wife-beating: understanding the pattern and trend in India

**DOI:** 10.1186/s12889-024-17782-w

**Published:** 2024-01-31

**Authors:** Manas Ranjan Pradhan, Prasenjit De

**Affiliations:** 1 Department of Fertility and Social Demography, International Institute for Population Sciences (IIPS), Govandi Station Road, Deonar, Mumbai, 400088 Maharashtra India; 2 International Institute for Population Sciences (IIPS), Govandi Station Road, Deonar, Mumbai, 400088 Maharashtra India

**Keywords:** Men, Attitude, Wife-beating, India, Trends

## Abstract

**Background:**

Intimate partner violence (IPV) is a severe human rights violation and a global burden on public health. Wife-beating is a form of IPV and an extension of the patriarchal philosophy that legitimizes men’s control over their spouses. This study investigates (a) the trends and patterns of men’s attitudes towards justification of wife-beating and (b) the socio-demographic factors associated with changes in men’s attitudes towards wife-beating between 2005–06 and 2019–21 in India.

**Methods:**

The present study utilized data from the last three rounds of the National Family Health Survey (NFHS): NFHS-3 (2005–06), NFHS-4 (2015–16), and NFHS-5 (2019–21) with a total sample of 2,76,672 men aged 15–54. The primary outcome variable was men’s attitudes toward wife-beating. Attitude towards the household and the sexual autonomy of the wife were the two key predictors, in addition to other structural factors. Descriptive, bivariate, and multivariate logistic regression analyses were performed on weighted data using Stata. Hosmer–Lemeshow test, Classification table, and ROC curve were carried out to enhance the robustness of the analysis and validity of the model.

**Results:**

In 2005–06, 50% of men justified wife-beating in at least one of the seven contexts, which reduced to 42% in 2015–16 and then marginally increased to 44% in 2019–21. Men with an authoritarian attitude toward household autonomy (AOR: 2.34; CI: 2.30,2.38) and sexual autonomy of the wife (AOR: 1.68; CI: 1.65,1.71) were more likely to justify wife-beating than their egalitarian counterparts. Inadequate education, younger age, family history of IPV, alcohol consumption, poverty, and rural settings are associated with an elevated risk of abusive attitudes towards wife-beating.

**Conclusion:**

A sizable percentage of men, more so those socio-economically marginalized, continue to justify wife-beating, albeit with considerable decline over the years. The findings suggest customized policies and programs enhancing gender egalitarian norms among young men, more opportunities to pursue higher education, alleviating poverty through employment opportunities, and raising awareness about domestic violence in rural settings would help develop more egalitarian gender norms and attitudes towards wife-beating.

**Supplementary Information:**

The online version contains supplementary material available at 10.1186/s12889-024-17782-w.

## Background

Intimate partner violence (IPV) is a severe violation of human rights and a global burden on public health [1–5]. IPV occurs regardless of social, cultural, and religious identities [6] and economic backgrounds [4]. Women carry most of the burden of IPV globally [6], with 15 to 71% experiencing physical or sexual abuse by their intimate partners [5, 7]. IPV against women has been associated with a plethora of immediate and long-term health consequences, including physical injuries, unwanted pregnancies, abortions, gynecological complications [8, 9], sexually transmitted infections, post-traumatic stress disorder, depression, and suicide [4, 10–12].

There are various forms of IPV, including wife-beating, which is commonly viewed as physical punishment by a husband to correct erred wife [13–15]. The wife-beating practice is an extension of the patriarchal philosophy that legitimizes the ideology that women are their spouses’ property [16]. One concerning aspect of wife-beating is its widespread social and cultural acceptance in many parts of the world [1]. IPV perpetration and social reaction to it are significantly influenced by attitudes that IPV is culturally acceptable [17, 18]. Responses to IPV are shaped by attitudes held by individuals other than those involved with the perpetration or victimization [17]. It is impossible to understand IPV behavior completely without comprehending the underlying attitudes [19]. Hence, understanding the attitudes toward wife-beating may be essential to comprehend the dynamics of wife-beating and designing effective interventions accordingly [14].

A growing body of literature tried to explore the attitudinal aspects of IPV against women. Attitude toward IPV is either based on the responses of only women [5, 20–22] or only men [16, 18, 23–25]. Some studies also attempted to incorporate the attitudes of both women and men, thereby explaining their differences [14, 17, 26]. Findings from these empirical studies explored individuals’ thinking about IPV, causes of justification of such violence, and potential risk factors that influence violence attitudes among individuals. Moreover, women’s attitude and the actual occurrence of IPV has been widely explored. Women who support their husband’s affirmative attitude toward violence are more likely to experience different forms of IPV than those who reject it [4, 27, 28]. Again, men’s attitudes toward violence were statistically significant in predicting violence between couples [29]. The documentation of the views of men on IPV is increasingly gaining attention from scholars and policymakers. Furthermore, empirical evidence on the attitude of men toward IPV is deemed useful in directing primary prevention initiatives to change societal perceptions and IPV norms [26, 30, 31].

## Theoretical background

The IPV is complex, multifaceted, and not confined to any particular theoretical aspect. Over the years, researchers have proposed several theoretical frameworks to explain the causes and dynamics of IPV. This study borrowed frameworks from Feminist theory [19, 32, 33], Social learning theory [34], and Ecological framework [35]. Feminist theory focuses on how gender-based power imbalances contribute to IPV [33, 36]. It suggests that IPV results from patriarchal values and beliefs where men use violence to control and maintain their dominant position over their partner [37]. Several authors empirically tested the validity of the Feminist theory to explain the complexity of IPV [38–40]. Given this particular theoretical background, the present study incorporates household decision-making autonomy and sexual autonomy (that also reflects the patriarchal views of male dominancy) as the main explanatory variables to predict the attitude toward wife-beating.

Initially developed by Bandura [41], Social learning theory suggests that IPV is learned through observation and imitation of violent behavior and may be more likely to occur in individuals exposed to violence in childhood [5]. In the context of IPV, individuals who have been exposed to violence in their family or community may learn that violence is an acceptable way to solve problems or exert control within a relationship. Previous literature suggests that persons who saw their fathers beat their mothers are likelier to develop violent behavior as an adult [5, 19, 42]. The present study uses the family history of violence as a predictor variable of men's attitudes towards wife-beating to determine the applicability of the social learning perspective in the Indian context.

**Fig. 1:**
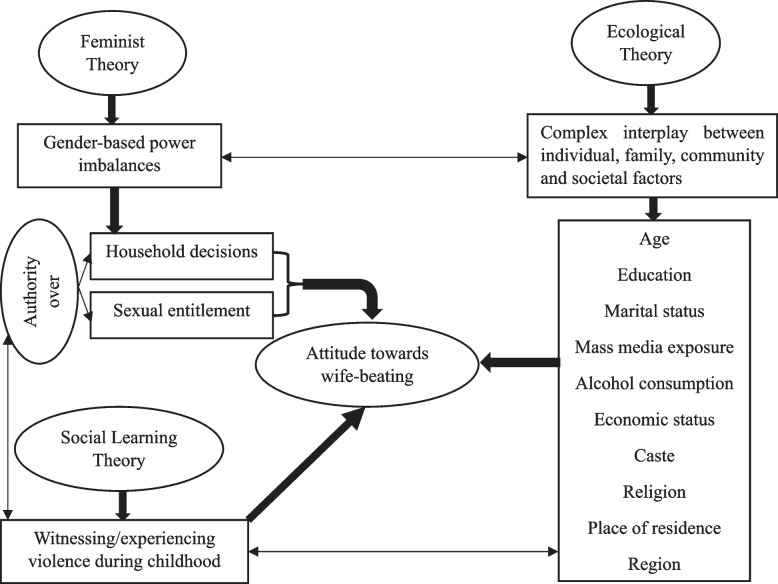
Predictors associated with men’s attitude towards wife-beating based on theoretical evidence

The ecological framework on partner abuse appears in the literature as a response to the drawbacks of Feminist theory and Social learning theory. Those theories fail to explain why certain men engage in physical abuse and sexual assault against women while others do not, despite being exposed to cultural norms that promote male dominance [35]. The ecological framework proposes that IPV results from a complex interplay between individual, family, community, and societal factors, and various levels within this societal structure can influence individual attitudes toward IPV [5]. This theory emphasizes the importance of considering the broader social and cultural context in which violence occurs. Based on the ecological framework, the present study incorporates socioeconomic variables like individuals’ age, educational attainment, caste, religious belief, exposure to mass media, alcohol use (personal/individual level factors), household wealth quintile (household/family level factors), place of residence, and region (community level factors). Figure 1 presents a conceptual framework related to predictors associated with men’s attitudes towards wife-beating based on these theoretical frameworks.

## Indian scenario

The IPV has become a serious concern in India [43, 44]. National Family Health Survey-5 (NFHS-5) found that 29.3% of ever-married women aged 18 to 49 years have ever experienced physical or sexual violence perpetrated by their intimate partners [45], while it was 31.2% during NFHS-4 [46]. Several studies have examined the prevalence and the potential risk factors related to IPV against women, types of behaviors that constitute IPV [43, 47, 48], and women’s attitudes towards violence [4, 22, 49]. Some studies also incorporated both women’s and men’s attitudinal aspects to understand the acceptability and actual perpetration of IPV against women [14, 19]. Although a substantial body of evidence exists from women’s perspective, only a limited number of studies have specifically investigated husbands' attitudes towards violence in the country [18, 37, 44].

Investigating the attitude toward wife-beating among men appears to be of utmost need for providing insights into its structural causes. However, empirical evidence on changing men’s attitudes toward violence against women over time is minimal. Few studies have specifically addressed the changing trends and patterns of men’s attitudes toward domestic violence [24] despite recognizing that this is an area of particular importance and warrants closer attention [1, 29]. The changing gender norms and societal acceptance of IPV over the years in the Indian scenario have also not been explored in detail in the scientific literature. To the authors’ knowledge, this study is the first to investigate the trends, patterns, and factors associated with changes in men’s perspectives regarding wife-beating using three rounds of nationally representative sample surveys in India. It will provide more insights into the discourse of IPV. Against this backdrop, this study investigates (a) the trends and patterns of men’s attitudes towards justification of wife-beating and (b) the socio-demographic factors associated with changes in men’s attitudes towards wife-beating between 2005–06 and 2019–21.

## Methods

### Source of data and study participants

**Fig. 2:**
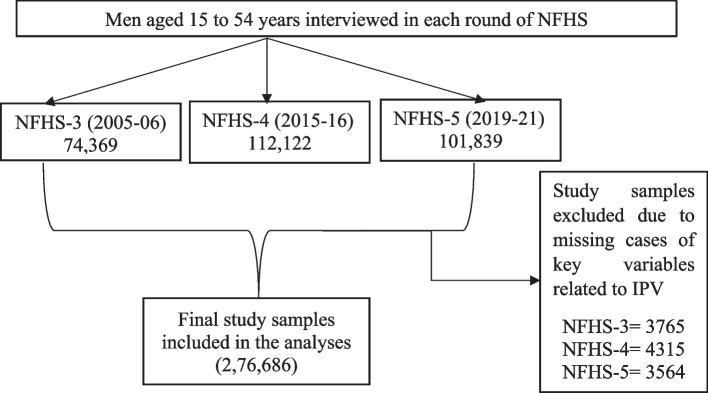
Flow charts of the selection of study participants

The study used data from the last three rounds of the NFHS-i.e., NFHS-3 (2005–06), NFHS-4 (2015–16), and NFHS-5 (2019–21). The NFHS is a large-scale, multi-round survey conducted in a representative sample of households throughout India. It gathered information on various health indicators from women aged 15–49 and men aged 15–54, including attitudes toward IPV. Informed consent procedures were followed, and only those who agreed voluntarily were interviewed by trained research investigators through Computer Assisted Personal Interview (CAPI). The round-specific survey reports include a minute description of the study design, sampling design, technique, and non-response rate [45, 46, 50]. The present study utilized data from Men’s files of all three rounds of NFHS. A total of 74,369, 112,122, and 101,839 men were interviewed during NFHS-3, NFHS-4, and NFHS-5, respectively. After eliminating missing values and ‘do not know’ cases of key variables for analysis purposes, the current study involved a total sample of 2,76,672 men aged 15–54 years (see Fig. 2). The data utilized in this study is available in the public domain and can be assessed through www.dhsprogram.com.

### Outcome variables

The primary outcome variable of this study was men’s attitudes toward wife-beating. It was assessed through their response to the following seven circumstances of justifying wife-beating: (a) if she goes out without telling, (b) neglects house or children, (c) argues with him, (d) refuses to have sex, (e) does not cook food properly, (f) suspected of being unfaithful, and (g) is disrespectful to in-laws. All the responses were converted into dichotomous: no (0), yes (1). For ease of analysis and more meaningful interpretation, the seven questions mentioned above were categorized into three categories as follows: Disagreement in opinion and mobility (goes out without telling and argues with him); Unfaithfulness (refuses to have sex and suspected of being unfaithful); and Neglects household chores and members (neglects house or children, does not cook food properly, and disrespectful to in-laws). While clubbing into categories, a value of 1 was assigned to men who justified wife-beating for any mentioned reasons nested within the category. In contrast, 0 was assigned to men who did not justify wife-beating for all the reasons within the category.

### Predictor variables

Men’s attitudes toward the wife’s autonomy in household decision-making and her sexual autonomy were the two principal predictor variables. Men’s attitude towards autonomy in household decision**s** was determined by asking men in a couple who should have a greater say (the husband, the wife, both equally) in making major household purchases, purchases for daily needs, visits to the wife’s family or relatives, what to do with the money the wife earns, and how many children to have. Men who said a wife should have an equal or greater say as her husband in any of the five specified decisions were considered to have an egalitarian attitude. In contrast, men who said only the husband should have the final say in all five decisions were considered to have an authoritarian attitude. Men with no opinion on the decisions above were recoded as unsure. Men were again asked if they think a wife is justified in refusing sex with her husband if she knows- he has a sexually transmitted disease, sex with other women, and she is tired or not in mode. Men who responded 'yes' to one or more of these circumstances were considered to have a more gender-egalitarian attitude. In contrast, men who disagreed were considered to have an authoritarian attitude, and men who did not have any opinion on the issues above were recorded as unsure.

Additionally, individual, household, and community-level variables, which could have potentially influenced the outcome variables, were also included in the analysis. Individual level predictors considered were men’s age group (15–24, 25–34, 35–44, 45–54 years); educational attainment (no education, primary, secondary, higher); marital status (never married, currently married, others [widowed, divorced no longer living together/separated]); mass media exposure (yes, if reads newspaper and/or magazines, listens to the radio, and watches television at least once a week or almost every day, no otherwise); and drinking alcohol (yes, no). Household level confounders included were caste (Scheduled Caste [SC], Scheduled Tribe [ST], Other Backward Classes [OBC], others [forward caste]); religion (Hindu, Muslim, Christian, others [Sikh, Buddhist, Jain, Jewish, no religion, and others]); family history of IPV (individuals were asked to answer the question of whether an individual’s father ever beat his mother. Responses were coded as yes, no, and do not know); and wealth quintile (poorest, poorer, middle, richer, richest [already given in the NFHS dataset]). Community level predictors included were the place of residence (urban, rural) and region (North, Central, East, North-East, West, South).

### Analytical approach

Descriptive statistics were performed to estimate men’s attitudes towards wife-beating on specific grounds. The bivariate percentage (weighted) of men in favor of wife-beating by the predictor variables was estimated using cross-tabulation. The intra-variable differences were tested using Pearson’s chi-square statistic across the survey rounds. Due to the dichotomous nature of the dependent variables, the multivariate logistic regression technique was used to evaluate the net effects of various explanatory variables on men’s attitudes towards wife-beating after controlling for other pertinent predictor variables. Two steps were involved in multivariate analysis to show the effects of predictors on changes in outcome variables over the year. First, binary logistic regression was performed separately with the same predictors for each survey wave. Then, the three waves were pooled to make a single dataset. A new variable, ‘time,’ which reflects each survey wave, was created to see the changes over the years. After adjusting the model to include the variable ‘time,’ the multivariable logistic regression was run on the pooled data to find significant predictors of men’s justification of wife-beating after controlling the extraneous influence of the survey rounds. The estimated adjusted odds ratio (AOR) with 95% confidence intervals (CI) was used to present the regression results. Multicollinearity among the predictor variables was examined through the Variance Inflation Factor (VIF) method. All the predictor variables used in the model had a VIF value below two, ruling out collinearity [51]. Regression model diagnostics such as the Hosmer–Lemeshow test, Classification table (indicating sensitivity, specificity, and overall accuracy of the model), and ROC curve that shows the overall accuracy of the regression model in predicting the outcome variable were carried out to enhance the robustness of the analysis and validity of the model. Additional file1 presents the classification table of logistic regressions depicting the overall accuracy of the model. All the statistical analyses were performed on weighted data using Stata version 17.0.

## Results

### Socio-demographic profile of the study population

Table 1 presents the distribution of the study population across socio-demographic characteristics. Of the total sample, 31% were aged 15–24, 28% were aged 25–34, 24% were aged 35–44, and the rest were aged 45–54. Fifty-six percent of the men were egalitarian, and 41% had an authoritarian attitude toward the wife’s autonomy in household decision-making. Two-thirds of the men possessed an egalitarian attitude towards the sexual autonomy of the wife. There was a consistent decline of men with no formal education over the study period- 19% in 2005–06, 13% in 2015–16, and 12% in 2019–21. About one-third (34%) of the sample were never married, and one-fifth (21%) had a family history of IPV. Caste-wise, men were almost evenly distributed across the survey rounds, and OBC constituted the highest proportion in every round. The proportion of Muslims increased from 12% in 2005–06 to 15% in 2019–21. The proportion of men who drink alcohol decreased over the survey rounds (from 32% in 2005–06 to 23% in 2019–21). The distribution of the study population by all other background characteristics did not vary significantly across the survey rounds.

**Table 1:** Socioeconomic and demographic profile of the respondents, India, 2005/06–2019-21

Background characteristics	NFHS-3 (2005–06)		NFHS-4 (2015–16)		NFHS-5 (2019–21)		All Rounds	
	**Weighted %**	**N**	**Weighted %**	**N**	**Weighted %**	**N**	**Weighted %**	**N**
**Attitude toward household autonomy**								
Egalitarian	50.42	37601	58.76	63214	56.6	59670	55.86	160485
Authoritarian	46.59	30685	38.67	41637	40.17	35637	41.23	107959
Unsure	2.99	2316	2.57	2956	3.23	2968	2.91	8240
**Attitude toward sexual autonomy of wife**								
Egalitarian	71.51	51957	64.06	69574	66.56	67409	66.85	160485
Authoritarian	20.57	13798	32.54	33775	30.97	28464	28.92	107959
Unsure	7.92	4836	3.4	4458	2.47	2402	4.23	8240
**Age group**								
15–24	32.78	23557	30.73	33440	29.13	29169	30.69	86166
25–34	28.06	20005	27.69	29913	27.19	26910	27.61	76828
35–44	23.63	16321	23.35	25175	23.87	23222	23.61	64718
45–54	15.52	10721	18.24	19279	19.81	18974	18.10	48974
**Education**								
No education	18.58	10195	13.08	14529	11.85	11812	14.05	36536
Primary	17.07	10907	12.65	13851	12.14	11379	13.60	36137
Secondary	51.76	38489	56.87	62436	56.61	57737	55.47	158662
Higher	12.59	10986	17.4	16991	19.39	17347	16.87	45324
**Marital status**								
Never married	33.16	26200	34.46	37603	34.83	34584	34.26	98387
Currently married	65.34	43456	64.13	68642	63.75	62186	64.30	174284
Others	1.5	948	1.41	1562	1.43	1505	1.44	4015
**Mass media exposure**								
No	6.83	3264	7.9	9976	12.22	14574	9.16	27814
Yes	93.17	67327	92.1	97831	87.78	83701	90.84	248859
**Drinking alcohol**								
No	67.61	46070	70.32	73825	76.92	72644	71.96	192539
Yes	32.39	24532	29.68	33982	23.08	25631	28.04	84145
**Caste**								
SC	18.86	12116	19.79	19318	20.27	18696	19.72	50130
ST	8.13	8140	8.75	18513	8.9	18101	8.64	44754
OBC	39.05	25557	43.48	42178	41.92	38265	41.80	106000
Others	33.96	24791	27.97	27798	28.92	23213	29.84	75802
**Religion**								
Hindu	82.14	52291	81.6	81049	79.51	75066	81.00	208406
Muslim	12.34	9169	13.16	14851	15.28	11719	13.70	35739
Christian	2.18	5914	2.18	6749	2.58	6482	2.32	19145
Others	3.33	3217	3.07	5158	2.63	5008	2.98	13383
**Family history of IPV**								
No	67.41	48206	76.43	83778	75.46	76024	73.78	208008
Yes	24.64	17490	20.04	19432	20.55	18512	21.40	55434
Do not know	7.95	4880	3.53	4597	3.99	3739	4.82	13216
**Wealth quintile**								
Poorest	15.87	6760	14.69	17781	16.54	18996	15.64	43537
Poorer	18.01	9723	18.65	22255	19.66	21738	18.84	53716
Middle	20.31	14050	21.06	23198	21.38	20961	20.98	58209
Richer	22.2	18377	22.23	22435	22.4	19577	22.28	60389
Richest	23.62	21694	23.38	22138	20.02	17003	22.25	60835
**Place of residence**								
Urban	36.73	36413	38.26	34189	35.36	25572	36.84	96174
Rural	63.27	34191	61.74	73618	64.64	72703	63.16	180512
**Region**								
North	14.39	8200	14.32	24031	8.59	20611	12.31	52842
Central	23.52	14962	21.83	27487	11.28	22614	18.53	65063
East	21.14	6374	18.85	16734	25.6	14836	21.83	37944
Northeast	3.83	11727	3.04	12815	5.31	13578	4.04	38120
West	15.73	10875	18.67	12123	24.27	11362	19.90	34360
South	21.39	18466	23.3	14617	24.96	15274	23.40	48357
**Total**	**100**	**70604**	**100**	**107807**	**100**	**98275**	**100**	**276686**

### Trends and patterns of attitude towards justification of wife-beating

**Fig. 3:**
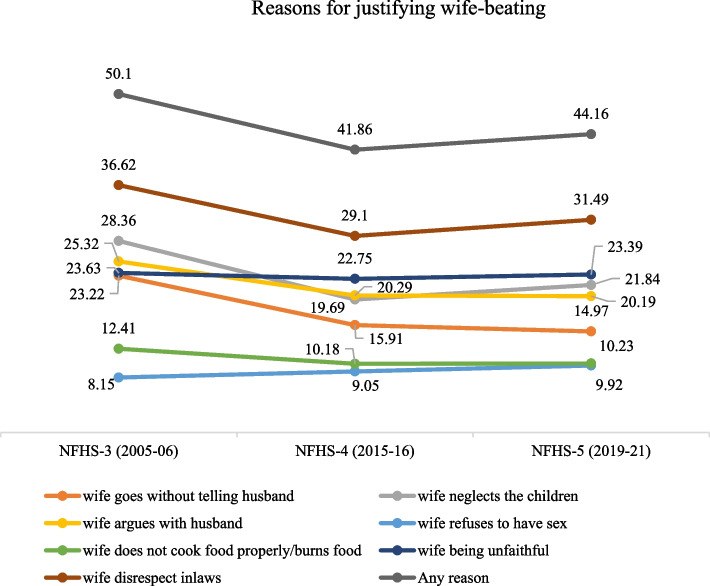
Percentage of men justified wife-beating by hypothetical reasons, India (2005–06 to 2019–21)

In 2005–06, 50% men justified wife-beating in at least one of the seven contexts, which reduced to 42% in 2015–16 and then marginally increased to 44% in 2019–21 (Fig. 3). In 2019–21, wife-beating was justified for disrespecting in-laws (31%), suspected unfaithfulness (23%), neglecting children (22%), opinion disagreement (20%), unauthorized mobility (15%), improper food and refusal to sex (10%). Compared to 2005–06, fewer men justified wife-beating based on disagreement in opinion and mobility, and neglect of household chores and members in 2019–21. However, the rate of justification of wife-beating for suspected unfaithfulness remained unchanged across the three survey rounds. Moreover, refusal to have sex as a reason for justifying wife-beating increased from 8% in 2005–06 to 9% in 2015–16 and again to 10% in 2019–21.

### Socioeconomic and demographic differentials of attitudes towards wife-beating

Nearly three-fifths (59%) of men with an authoritarian attitude in household decision-making justified wife-beating for at least one listed reason compared to one-third (34%) of their counterparts with an egalitarian attitude (Table 2). A similar situation was found for disagreement in opinion and mobility, unfaithfulness, and negligence to household chores and members in all survey rounds. Wife-beating justification was higher among men with an authoritarian attitude towards the sexual autonomy of their wives (57%) compared with their peers with an egalitarian attitude (38%). Justification of wife-beating decreased with increasing age across all the specific reasons over the survey rounds. Affirmative attitudes towards wife-beating decreased with the increased educational attainment of the men. Of the never-married men, 47% justified wife-beating for at least one of the reasons compared with 44% of those currently married. A considerably higher proportion of backward caste men favored wife-beating than those from forward caste across all the survey rounds. A higher proportion of Muslim men justified wife-beating than Hindus for all the specified reasons. A higher proportion of men who consumed alcohol, those who resided in rural settings, and those with a family history of IPV justified wife-beating across all the specified reasons. Fifty percent of the men from the poorest wealth quintile justified wife-beating compared to 34% of those from the richest quintile. Two-thirds of the men from the southern region justified wife-beating for at least one of the reasons. The corresponding figures were 35% in the north, 36% in the northeast, 37% in the west, 38% in the east, and 42% in the central region.

**Table 2:** Men’s justification of wife-beating for the specified reasons by background characteristics, India, 2005/06–2019-21

Predictors	Justified at least one reason				Disagreement in opinion and mobility				Unfaithfulness				Neglects household chores and members			
	**Weighted percentage (%) of N**				**Weighted percentage (%) of N**				**Weighted percentage (%) of N**				**Weighted percentage (%) of N**			
	**NFHS-3** **(2005–06)**	**NFHS-4** **(2015–16)**	**NFHS-5** **(2019–21)**	**All** **Rounds**	**NFHS-3** **(2005–06)**	**NFHS-4** **(2015–16)**	**NFHS-5** **(2019–21)**	**All Rounds**	**NFHS-3** **(2005–06)**	**NFHS-4** **(2015–16)**	**NFHS-5** **(2019–21)**	**All Rounds**	**NFHS-3** **(2005–06)**	**NFHS-4** **(2015–16)**	**NFHS-5** **(2019–21)**	**All Rounds**
**Attitude toward household autonomy**																
Egalitarian	38.82	32.28	34.09	34.44	23.95	17.51	17.69	19.06	18.72	18.53	19.50	18.92	33.40	27.01	29.42	29.35
Authoritarian	61.86	56.06	58.36	58.53	44.55	36.95	36.38	38.95	33.26	35.69	37.14	35.49	55.18	47.27	51.03	50.85
Unsure	56.98	47.16	44.09	48.54	39.85	28.68	24.31	29.90	35.64	28.45	27.96	30.15	50.15	40.27	37.96	41.96
**Attitude toward sexual autonomy of wife**																
Egalitarian	45.60	36.24	38.22	39.50	30.75	21.08	20.48	18.92	22.65	22.13	22.62	22.45	39.79	30.48	33.27	34.01
Authoritarian	62.68	52.33	56.64	55.85	42.46	32.92	35.74	35.49	34.93	31.24	35.79	33.64	56.56	43.91	49.18	48.21
Unsure	58.00	47.35	47.82	52.55	41.61	32.28	28.80	30.15	32.99	31.69	29.23	31.80	49.94	40.27	40.52	44.95
**Age Group**																
15–24	55.46	55.55	45.36	47.77	37.61	27.89	26.75	30.16	29.01	27.04	27.64	27.78	49.45	37.82	39.56	41.58
25–34	49.63	58.41	43.86	44.48	33.68	24.38	25.17	27.07	25.11	25.06	26.09	25.43	43.55	35.10	38.37	38.44
35–44	46.69	59.86	43.14	42.89	31.97	24.17	24.43	26.26	24.83	24.12	26.20	25.05	40.47	33.37	36.92	36.46
45–54	44.81	59.93	44.06	42.66	30.17	23.86	24.98	25.68	23.02	24.91	27.59	25.53	39.01	33.19	38.41	36.49
**Education**																
No education	61.18	48.26	50.08	53.17	46.26	31.96	31.19	36.57	36.16	31.89	31.48	33.21	53.82	40.38	43.82	45.96
Primary	58.32	45.03	48.97	50.54	41.56	28.90	30.04	33.32	31.91	28.48	29.79	30.00	51.20	37.31	42.19	43.31
Secondary	48.49	41.73	44.49	44.34	31.86	24.89	25.58	26.80	23.83	24.75	27.22	25.42	42.75	35.17	38.70	38.25
Higher	29.20	35.14	36.59	34.60	14.57	19.09	18.49	17.98	11.89	20.51	21.18	19.14	25.30	29.80	31.70	29.72
**Marital status**																
Never married	52.40	44.30	45.46	46.72	34.52	27.26	26.09	28.64	26.65	27.09	27.73	27.21	46.93	37.76	40.05	40.86
Currently married	48.75	40.45	43.39	43.64	33.51	24.12	24.92	26.84	25.39	24.43	26.28	25.33	42.46	33.71	37.40	37.28
Others	58.04	45.87	46.85	49.45	45.14	31.93	30.97	35.11	38.15	29.90	31.81	32.76	49.56	39.60	41.07	42.77
**Mass media exposure**^**a**^																
No	61.19	44.80	42.18	46.69	44.29	30.74	26.05	31.11	37.37	28.76	25.40	28.82	52.95	37.70	35.64	39.64
Yes	49.29	41.60	44.44	44.59	33.27	24.85	25.32	27.22	25.17	25.13	27.07	25.80	43.40	34.97	38.76	38.48
**Drinking alcohol**																
No	48.20	38.41	42.05	42.14	32.21	22.84	23.81	25.46	24.51	22.68	25.57	24.21	42.55	32.35	36.35	36.32
Yes	54.05	50.01	51.20	51.55	37.79	31.18	30.75	33.01	29.10	31.90	31.18	30.87	47.19	41.89	45.14	44.40
**Caste**																
SC	53.89	45.98	44.34	47.32	37.58	29.23	27.03	30.47	28.45	28.06	26.38	27.54	47.46	39.15	38.95	41.11
ST	58.64	43.20	43.50	47.03	41.04	27.12	25.18	29.76	35.59	28.61	26.22	29.42	51.76	35.52	37.70	40.22
OBC	53.66	44.44	46.97	47.54	37.91	26.97	26.24	29.33	27.77	27.40	28.58	27.91	47.07	37.47	41.34	41.14
Others	41.85	34.50	40.18	38.59	25.90	19.40	23.15	22.58	20.31	19.47	24.91	21.58	36.84	28.72	33.89	32.86
**Religion**																
Hindu	50.09	41.56	43.47	44.44	34.03	25.10	25.00	27.39	25.79	25.21	26.11	25.67	44.14	34.93	38.04	38.40
Muslim	52.95	42.68	47.85	47.09	36.87	26.69	28.45	29.73	28.52	26.26	30.77	28.56	46.25	35.69	40.34	39.96
Christian	48.70	52.44	55.27	52.65	28.32	27.96	29.33	28.59	27.25	33.68	38.66	34.09	42.50	46.18	47.63	45.86
Others	40.79	38.58	32.75	37.39	27.01	23.11	16.31	22.10	20.98	21.62	15.48	19.52	34.87	31.90	28.28	31.62
**Family history of IPV**																
No	44.75	36.42	37.95	38.92	29.02	21.39	20.54	22.86	23.21	21.49	22.86	22.39	39.01	30.31	32.24	33.04
Yes	64.81	61.32	65.90	63.91	47.36	39.78	42.54	42.95	33.62	40.10	40.38	38.29	58.21	52.63	59.89	56.74
Do not know	49.79	49.05	49.77	49.57	35.06	28.19	29.32	31.42	25.96	27.18	32.98	28.36	42.88	41.75	43.72	42.80
**Wealth index**																
Poorest	60.17	45.94	46.21	49.73	46.27	31.88	29.31	34.65	36.23	29.14	28.54	30.75	52.71	37.86	38.51	41.96
Poorer	58.52	43.03	45.94	47.89	42.95	27.85	27.69	31.48	31.98	26.26	27.79	28.23	51.39	35.63	39.57	40.94
Middle	56.84	45.09	47.21	48.76	38.75	27.17	26.89	29.94	29.71	27.80	28.67	28.58	50.32	38.27	42.08	42.63
Richer	47.94	43.70	45.74	45.51	30.13	24.61	25.12	26.20	22.88	26.56	27.94	26.12	42.27	37.12	40.31	39.57
Richest	33.14	33.69	35.72	34.19	18.57	18.15	18.71	18.44	14.30	19.19	21.43	18.57	28.93	28.53	30.98	29.42
**Place of residence**																
Urban	41.32	38.30	40.37	39.77	25.03	22.17	22.02	22.85	18.28	22.58	24.23	22.04	36.54	32.83	35.42	34.65
Rural	55.20	44.06	46.24	47.70	39.24	27.26	27.27	30.33	30.48	27.18	28.31	28.43	48.41	36.65	39.99	40.88
**Region**																
North	46.70	31.93	27.94	35.36	32.55	19.45	15.87	22.48	29.26	18.77	16.58	21.37	39.24	27.17	23.57	29.89
Central	47.25	41.26	35.42	41.95	34.65	27.19	21.08	28.30	25.35	25.68	21.00	24.57	41.00	33.39	29.41	35.01
East	43.56	33.34	39.51	38.44	29.71	21.89	25.21	25.21	25.14	18.76	24.92	22.90	35.13	25.66	31.57	30.46
Northeast	34.47	36.58	35.97	35.79	20.40	24.26	17.25	20.07	16.98	23.71	19.60	20.17	29.72	29.87	29.61	29.72
West	54.26	31.67	33.25	36.92	32.80	15.40	16.22	19.27	19.58	15.28	16.61	16.72	52.08	27.78	29.04	33.24
South	61.72	64.25	70.83	66.14	41.91	38.01	41.53	40.25	31.70	43.00	46.57	41.70	56.12	56.12	65.45	59.64

^a^Pearson chi-square statistics reveal that all values of weighted % are statistically significant (*p* < 0.001) except the bivariate distribution of mass-media exposure and men's justification of wife beating by reason of sensitive interpersonal disputes in NFHS-5 (*p* > 0.05)

## Determinants of men’s attitude towards justification of wife-beating

Multivariate logistic regression revealed that controlling for the influence of the survey rounds and other predictors, men with an authoritarian attitude regarding household decision-making were 2.34 times (AOR: 2.34; CI: 2.30,2.38) and those unsure were 1.52 times (AOR: 2.3; 95% CI: 1.45,1.59) more likely to justify wife-beating for at least one of the listed reasons compared to men with an egalitarian attitude (Table 3). Men with a final say in household decisions were 2.28 times (AOR: 2.28; CI: 2.20,2.35), 2.37 times (AOR: 2.37; CI: 2.30,2.43), and 2.39 times (AOR: 2.39; CI: 2.32,2.46) more likely to justify wife-beating than men with an egalitarian attitude in 2005–06, 2015–16, and 2019–21, respectively. Men with an authoritarian attitude towards the sexual autonomy of the wife were 68% (AOR: 1.68; CI: 1.65,1.71) more likely to justify wife-beating than men with an egalitarian attitude. In 2005–06, men with an authoritarian attitude towards the sexual autonomy of the wife were 62% (AOR: 1.62; CI: 1.55,1.68) more likely to justify wife-beating, which came down to 56% (AOR: 1.56; CI: 1.52,1.60) in 2015–16 but increased to 78% (AOR: 1.78; CI: 1.72,1.83) in 2019–21. Men aged 45–54 had the lowest odds of justifying wife-beating except in 2019–21. Men with secondary education (AOR: 0.81, CI: 0.79, 0.84) and higher education (AOR: 0.63, CI: 0.61, 0.65) were less likely to justify wife-beating than uneducated men. This pattern was consistent across survey rounds. Men in a marital union were significantly less likely to justify wife-beating than never-married men except in 2005–06. Men who consume alcohol were 25% (AOR: 1.25; CI: 1.22,1.27) more likely to justify wife-beating than their non-alcoholic counterparts, and this relationship persisted in other survey rounds. Men from the forward caste group were less likely to justify wife-beating than the SC category, except in 2019–21 (AOR: 1.14; CI: 1.09,1.19), where they were more likely to justify wife-beating. However, no consistent pattern emerged in the association between other caste groups and men’s attitudes towards wife-beating. Compared to Hindus, Muslim men had higher odds (AOR: 1.24; CI: 1.21, 1.27) of justifying wife-beating, and this pattern was consistent across all survey rounds. Men with a family history of IPV had higher odds (AOR: 1.96; CI: 1.92,2.00) of justifying wife-beating than their counterparts. Men in the wealthiest households were 37% (AOR: 0.63; CI: 0.61,0.65) less likely to justify wife-beating than those from the poorest households in all years.

**Table 3:** Multivariable logistic regression result for justifying wife-beating for at least one reason and unfaithfulness by men in India, 2005/06–2019-21

Predictors	Justified At least One reason				Unfaithfulness			
	**All Years**	**NFHS-3** **(2005–06)**	**NFHS-4** **(2015–16)**	**NFHS-5** **(2019–21)**	**All Years**	**NFHS-3** **(2005–06)**	**NFHS-4** **(2015–16)**	**NFHS-5** **(2019–21)**
	**AOR [95% CI]**	**AOR [95% CI]**	**AOR [95% CI]**	**AOR [95% CI]**	**AOR [95% CI]**	**AOR [95% CI]**	**AOR [95% CI]**	**AOR [95% CI]**
**Attitude toward household autonomy**								
Egalitarian®								
Authoritarian	2.34*** [2.30,2.38]	2.28*** [2.20,2.35]	2.37*** [2.30,2.43]	2.39*** [2.32,2.46]	2.04*** [2.00,2.07]	1.84*** [1.78,1.91]	2.11*** [2.04,2.17]	2.10*** [2.03,2.16]
Unsure	1.52*** [1.45,1.59]	1.78*** [1.62,1.96]	1.54*** [1.42,1.68]	1.41*** [1.31,1.53]	1.58*** [1.50,1.67]	2.08*** [1.89,2.30]	1.43*** [1.30,1.56]	1.47*** [1.35,1.60]
**Attitude toward sexual autonomy of wife**								
Egalitarian®								
Authoritarian	1.68*** [1.65,1.71]	1.62*** [1.55,1.68]	1.56*** [1.52,1.60]	1.78*** [1.72,1.83]	1.46*** [1.44,1.49]	1.53*** [1.46,1.59]	1.26*** [1.22,1.30]	1.56*** [1.51,1.61]
Unsure	1.23*** [1.18,1.28]	1.19*** [1.12,1.27]	1.15*** [1.07,1.23]	1.20*** [1.10,1.31]	1.25*** [1.20,1.30]	1.23*** [1.16,1.31]	1.18*** [1.09,1.27]	1.14** [1.04,1.26]
**Age group**								
15–24®								
25–34	0.86*** [0.84,0.88]	0.75*** [0.71,0.79]	0.89*** [0.85,0.93]	0.95* [0.91,1.00]	0.88*** [0.85,0.91]	0.79*** [0.74,0.83]	0.91*** [0.87,0.96]	0.95* [0.90,1.00]
35–44	0.76*** [0.74,0.79]	0.62*** [0.59,0.66]	0.83*** [0.78,0.87]	0.86*** [0.81,0.90]	0.83*** [0.80,0.85]	0.73*** [0.68,0.78]	0.86*** [0.81,0.91]	0.91** [0.86,0.97]
45–54	0.74*** [0.72,0.77]	0.57*** [0.53,0.61]	0.78*** [0.74,0.82]	0.90*** [0.85,0.95]	0.82*** [0.79,0.85]	0.64*** [0.60,0.69]	0.85*** [0.80,0.90]	0.97 [0.92,1.04]
**Education**								
No education®								
Primary	0.95*** [0.92,0.98]	0.89*** [0.84,0.94]	0.94* [0.89,0.99]	1.07* [1.01,1.13]	0.92*** [0.89,0.95]	0.91*** [0.86,0.96]	0.92** [0.87,0.97]	1.03 [0.97,1.09]
Secondary	0.81*** [0.79,0.84]	0.70*** [0.66,0.74]	0.87*** [0.83,0.91]	0.95* [0.90,0.99]	0.79*** [0.77,0.82]	0.72*** [0.69,0.76]	0.81*** [0.77,0.85]	0.97 [0.92,1.02]
Higher	0.63*** [0.61,0.65]	0.45*** [0.42,0.49]	0.70*** [0.66,0.74]	0.74*** [0.70,0.79]	0.63*** [0.60,0.65]	0.46*** [0.42,0.50]	0.68*** [0.64,0.72]	0.74*** [0.70,0.79]
**Marital status**								
Never married®								
Currently married	0.91*** [0.88,0.93]	0.96 [0.91,1.01]	0.85*** [0.81,0.89]	0.88*** [0.84,0.92]	0.90*** [0.88,0.93]	0.97 [0.91,1.02]	0.83*** [0.79,0.87]	0.88*** [0.84,0.93]
Others	1.03 [0.96,1.10]	1.23** [1.06,1.41]	1 [0.89,1.12]	0.91 [0.80,1.03]	1.18*** [1.10,1.27]	1.53*** [1.33,1.76]	1.02 [0.90,1.16]	1.08 [0.95,1.23]
**Mass media exposure**								
No®								
Yes	1.04** [1.01,1.08]	0.95[0.89,1.02]	0.93** [0.89,0.98]	1.11*** [1.06,1.17]	1.03 [1.00,1.07]	0.94 [0.88,1.01]	0.90*** [0.85,0.96]	1.11*** [1.06,1.17]
**Drinking alcohol**								
No®								
Yes	1.25*** [1.22,1.27]	1.19*** [1.15,1.24]	1.35*** [1.31,1.39]	1.18*** [1.14,1.22]	1.19*** [1.16,1.21]	1.16*** [1.12,1.21]	1.30*** [1.26,1.35]	1.08*** [1.04,1.12]
**Caste**								
SC®								
ST	1 [0.97,1.04]	1.11** [1.03,1.19]	0.96 [0.91,1.01]	1.01 [0.95,1.07]	1.15*** [1.11,1.19]	1.27*** [1.18,1.36]	1.13*** [1.07,1.20]	1.07* [1.01,1.14]
OBC	1 [0.98,1.02]	1.08*** [1.03,1.13]	0.93*** [0.89,0.96]	1.03 [0.99,1.07]	1.01 [0.99,1.04]	1.07** [1.02,1.13]	0.97 [0.93,1.01]	1.02 [0.97,1.06]
Others	0.96** [0.94,0.99]	0.88*** [0.83,0.92]	0.88*** [0.85,0.92]	1.14*** [1.09,1.19]	0.99 [0.96,1.02]	0.88*** [0.83,0.93]	0.90*** [0.86,0.94]	1.19*** [1.13,1.25]
**Religion**								
Hindu®								
Muslim	1.24*** [1.21,1.27]	1.22*** [1.16,1.28]	1.19*** [1.15,1.24]	1.28*** [1.22,1.33]	1.26*** [1.22,1.29]	1.25*** [1.18,1.32]	1.21*** [1.16,1.27]	1.30*** [1.24,1.36]
Christian	1.14*** [1.07,1.20]	1.04 [0.93,1.17]	1.15** [1.05,1.25]	1.21*** [1.11,1.33]	1.25*** [1.18,1.32]	1.25*** [1.10,1.41]	1.10* [1.00,1.21]	1.45*** [1.32,1.58]
Others	1.10*** [1.05,1.16]	0.85*** [0.77,0.93]	1.36*** [1.26,1.47]	1.08 [0.99,1.18]	1.04 [0.98,1.10]	0.90* [0.80,1.00]	1.29*** [1.18,1.41]	0.9 [0.81,1.01]
**Family history of IPV**								
No®								
Yes	1.96*** [1.92,2.00]	1.76*** [1.70,1.84]	1.90*** [1.84,1.96]	2.19*** [2.11,2.27]	1.55*** [1.52,1.58]	1.33*** [1.28,1.39]	1.66*** [1.61,1.72]	1.58*** [1.53,1.64]
Do not know	1.47*** [1.41,1.52]	1.13*** [1.07,1.20]	1.73*** [1.62,1.86]	1.60*** [1.50,1.72]	1.32*** [1.26,1.37]	1.04 [0.98,1.11]	1.31*** [1.21,1.41]	1.58*** [1.47,1.70]
**Wealth Index**								
Poorest®								
Poorer	0.88*** [0.86,0.91]	0.97 [0.92,1.03]	0.85*** [0.81,0.89]	0.87*** [0.83,0.91]	0.87*** [0.85,0.90]	0.93* [0.88,0.98]	0.85*** [0.81,0.89]	0.86*** [0.82,0.91]
Middle	0.82*** [0.80,0.85]	0.89*** [0.84,0.95]	0.83*** [0.79,0.87]	0.78*** [0.74,0.82]	0.83*** [0.80,0.86]	0.87*** [0.82,0.93]	0.83*** [0.79,0.88]	0.79*** [0.75,0.83]
Richer	0.77*** [0.75,0.80]	0.73*** [0.68,0.77]	0.82*** [0.78,0.86]	0.77*** [0.73,0.81]	0.80*** [0.78,0.83]	0.76*** [0.71,0.81]	0.83*** [0.78,0.88]	0.80*** [0.75,0.85]
Richest	0.63*** [0.61,0.65]	0.55*** [0.52,0.60]	0.69*** [0.65,0.73]	0.64*** [0.60,0.68]	0.68*** [0.65,0.71]	0.61*** [0.56,0.66]	0.69*** [0.65,0.74]	0.71*** [0.67,0.76]
**Place of residence**								
Urban®								
Rural	1.19*** [1.16,1.21]	1.28*** [1.23,1.33]	1.15*** [1.12,1.19]	1.17*** [1.13,1.21]	1.20*** [1.18,1.23]	1.38*** [1.32,1.44]	1.14*** [1.10,1.18]	1.16*** [1.11,1.20]
**Region**								
North®								
Central	1.04* [1.00,1.07]	0.72*** [0.69,0.77]	1.24*** [1.18,1.29]	1.15*** [1.08,1.23]	0.97 [0.93,1.00]	0.61*** [0.58,0.65]	1.22*** [1.16,1.29]	1.14*** [1.06,1.23]
East	0.78*** [0.76,0.81]	0.51*** [0.49,0.55]	0.81*** [0.77,0.85]	1.19*** [1.12,1.26]	0.77*** [0.75,0.80]	0.52*** [0.49,0.56]	0.77*** [0.72,0.81]	1.23*** [1.15,1.32]
Northeast	0.78*** [0.74,0.82]	0.42*** [0.38,0.46]	0.96 [0.88,1.04]	1.15*** [1.06,1.24]	0.69*** [0.66,0.73]	0.36*** [0.32,0.40]	1.06 [0.97,1.17]	0.96 [0.87,1.05]
West	1.12*** [1.08,1.15]	1.42*** [1.34,1.51]	1.10*** [1.05,1.16]	1.26*** [1.19,1.34]	0.73*** [0.70,0.75]	0.57*** [0.54,0.61]	0.85*** [0.80,0.90]	0.97 [0.91,1.04]
South	2.80*** [2.71,2.88]	1.37*** [1.29,1.45]	3.05*** [2.91,3.20]	4.80*** [4.52,5.10]	2.05*** [1.98,2.12]	0.87*** [0.82,0.93]	2.65*** [2.51,2.79]	3.45*** [3.23,3.69]
**Time**								
NFHS-3 (2005–06)®								
NFHS-4 (2015–16)	0.74*** [0.72,0.75]	-	-	-	1.05*** [1.02,1.07]	-	-	-
NFHS-5 (2019–21)	0.82*** [0.80,0.84]	-	-	-	1.16*** [1.13,1.19]	-	-	-
N	276,686	70,604	107,807	98,275	276,686	70,604	107,807	98,275
Pseudo R^2^	0.127	0.301	0.115	0.146	0.225	0.185	0.143	0.105
Pearson's chi-squared goodness-of-fit test	0.115	0.404	0.070	0.151	0.163	0.112	0.137	0.183

® Reference category; 95% confidence intervals in parentheses, *AOR* Adjusted Odds Ratio

^*^p < 0.05

^**^p < 0.01

^***^p < 0.001

In the pooled dataset, men in rural areas were more likely to justify wife-beating than their urban counterparts (AOR: 1.19; CI: 1.16, 1.21), and this pattern persisted in all survey rounds. Men from the southern region were 2.80 times (AOR: 2.80; CI: 2.71,2.88) more likely to justify wife-beating than their northern counterparts. Men were 26% (AOR: 0.74; CI: 0.72,0.75) and 18% (AOR: 0.82; CI: 0.80,0.84) less likely to justify wife-beating for at least one of the reasons during 2015–16 and 2019–21, respectively, compared to 2005–06.

Men’s justification for wife-beating remained unchanged for ‘wife suspected of being unfaithful’ and increased for ‘sexual refusal’ from 2005–06 to 2019–21, urging the need to examine predictors’ effects over time. Men with an authoritarian attitude in household decision-making had more than two times higher odds of justifying wife-beating for unfaithfulness (AOR: 2.04; CI: 2.00,2.07) than men with egalitarian attitudes. Again, men with an authoritarian attitude toward the sexual autonomy of the wife were more (AOR: 1.46; CI: 1.44,1.49) likely to justify wife-beating for unfaithfulness than those with an authoritarian attitude in the pooled dataset. A similar pattern also persisted for all the survey rounds. Men aged 45–54 were less likely (AOR: 0.82; CI: 0.79,0.85) to justify wife-beating for unfaithfulness than those aged 15–24. Higher educated men had the lowest odds of justifying wife-beating for unfaithfulness than their non-literate counterparts. Men in a marital union were significantly less likely to justify wife-beating for unfaithfulness than never-married men. Alcohol consumption was associated with a higher likelihood of justifying wife-beating for unfaithfulness for all the survey years. Muslim and Christian men were likelier to justify wife-beating for unfaithfulness than their Hindu counterparts. The likelihood of men justifying wife-beating for unfaithfulness was greater (AOR: 1.55; CI: 1.52,1.58) among those with a family history of IPV than those without a family history of IPV. This pattern persisted across all survey rounds. Men belonging to the wealthier strata had lower odds of justifying wife-beating for unfaithfulness than their poorest counterparts. Men in rural areas were more likely to justify wife-beating for unfaithfulness than their urban counterparts. Men from the south region were more than two times more likely to justify wife-beating for unfaithfulness (AOR: 2.05; CI: 1.98,2.12) than their northern counterparts. The odds of justifying wife-beating for unfaithfulness increased during 2019–21 (AOR: 1.16; CI: 1.13,1.19) compared to 2005–06.

## Discussion

The justification for wife-beating has considerably declined over the years, though a sizable percentage of men continue to justify it in several circumstances. From 2005/06 to 2019/21, men’s justification for wife-beating for suspected unfaithfulness has remained unchanged and has increased for her refusal to have sex. Disrespecting in-laws, followed by suspected unfaithfulness, are the primary reasons for justifying wife-beating. Attitude toward wife-beating is highly influenced by men’s attitude toward household decision-making and the sexual autonomy of women. Other significant predictors of wife-beating justification are age structure, education, alcohol consumption, family history of IPV, wealth strata, place of residence, and geographical region.

The study found that more than two-fifths of the men justify wife-beating in 2019–21, and the finding conforms to several recent studies in Africa [24, 52] and Asia [16]. Men in India continue to justify wife-beating for suspected unfaithfulness, which may be attributed to the strong cultural norms of male superiority to women [42] and also a substantial proportion of men being unwilling to relinquish their traditional gender roles that are stereotypically associated with their sex [14]. It is further found that men think women should have minimal authority over their sexuality [44]. Moreover, the increasingly intolerant attitude of men on the ground that the wife refuses to have sex may be attributable to the interviewer effect due to the high sensitivity of these questions. An earlier study based on women’s samples covered in the NFHS found a significant interviewer effect in the trend of wife-beating justification related to a woman’s refusal to have sex with her husband [53].

Men with an egalitarian attitude in household decision-making were significantly less likely to justify wife-beating. A past study reveals that mutual decisions regarding daily household purchases afford the wife greater protection [44]. Men who disapprove of women’s sexual autonomy were more likely to justify wife-beating for all the reasons across the three waves of the survey. These results corroborate findings from past studies on homogeneous settings [4, 14, 19, 37]. The findings of the present study show that male supremacy, women subjugation, and the controlling behavior of men in a marital relationship are still relevant in the Indian scenario. It emphasizes the necessity of regular and reciprocal communication to alleviate the husband’s tolerant attitudes regarding wife-beating [44].

This study found that men with a family history of IPV were more likely to justify abusive behaviors toward their wives. This finding underscores the importance of social learning theory, which suggests that domestic violence can be transmitted across generations through social learning [44]. It also aligns with previous research conducted in similar as well as diverse geographical and cultural contexts, which has established that witnessing parental violence during childhood constitutes a significant risk factor for perpetrating IPV in adulthood [22, 26, 42, 44].

The study found younger men with less tolerant attitudes toward wife-beating. One plausible explanation may be that older men possess a greater understanding of dyadic relationships, resulting in a decreased justification towards wife-beating compared to younger men with limited exposure to such relationships [24]. Men’s tolerant attitude towards wife-beating showed an inverse relationship with their education level and household wealth quintile. Many studies from the developing world also established similar findings [14, 15, 43, 44, 52]. A conceivable rationale for these findings is that men who have received higher education and those residing in households belonging to the uppermost wealth quintile are more likely to be exposed to modern and egalitarian gender norms than their counterparts [15]. The negative relationship between household economic status and justification of wife-beating further confirms that socioeconomic deprivation and inequalities also play an important role in developing violent behavior besides the patriarchic gender norms. The relationship between poverty and IPV is mediated through stress, as poverty, inherently stressful, is considered a potential factor contributing to IPV [54]. Another view is that a person with low economic resources is more likely to develop violent behavior to attain power and dominance over intimate relations, as it is the only available resource to utilize [55].

Results show that more men who consume alcohol justify wife-beating for all specified reasons. Alcohol has been proven to be a disinhibiting agent in certain forms of sexual assault [35]. In the present study, currently married men were less likely to justify wife-beating than those who never married, which conforms to an earlier study in a similar setting [44]. This could be attributed to men’s better understanding of the dyadic relationships and power dynamics within marital unions [15]. The study found that men exposed to mass media are more likely to justify wife-beating in the most recent round of the survey, which conforms to a past study [18]. In agreement with an earlier study, this study also found lower chances of wife-beating justification among men from the forward caste [18]. Muslim men were significantly more likely to justify wife-beating than their Hindu counterparts, and this result agrees with prior studies [14, 15, 56].

Several studies have consistently found that men living in rural settings are significantly more likely to justify wife-beating [14, 24, 37], as found in the present study. This could be explained by the prevalent egalitarian gender norms within urban households [57], as urbanites are more exposed to a modern culture where gender equals norms are more fashionable [15]. Men from the southern region of India justify wife-beating more than their northern counterparts over the years, except for unfaithfulness in 2005–06. This finding is unusual and not in line with prior studies, as female powerlessness is much more pronounced in north India, and women from south India enjoy relatively more autonomy [13] and more egalitarian norms [58]. Another study also found that various areas of northern India are more or less in favor of gender-based violence [42]. A more detailed qualitative study would be required to explore variations within the region in men’s abusive attitudes toward their wives.

### Limitations and strengths

Due to the cross-sectional nature of the survey, it is impossible to establish a causal relationship between the justifications for wife-beating by men and the predictors used in this study. Another methodological constraint is that men (study samples) tend to underreport wife-beating justification due to the sensitive nature of the questions [42, 44], which may hamper the actual statistics. The study was limited to men’s perception of wife-beating justification by seven hypothetical circumstances. An empirical investigation of endogeneity between justification and experience of violence is beyond the scope of the present study. Some psychological research has demonstrated that the association between attitudes and actual behavior may exhibit a robust correlation only sometimes [37]. Another limitation is that it failed to investigate the interviewer’s effects on sensitive questions of sexuality and wife-beating justification, which could influence the study’s outcome. There is again the possibility of other cultural and contextual factors influencing the attitude towards wife-beating, which could not be included due to data unavailability. Considering the study’s limitation, there is a need for longitudinal studies examining changes in the trends and patterns of wife-beating attitudes at the individual level. Furthermore, a qualitative investigation is needed to provide more insights into the continuing justification of wife-beating on specific grounds among Indian men. Despite these limitations, the present study provides insightful findings on trends, patterns, and associated factors of abusive attitudes of men toward wife-beating in the Indian context. To our knowledge, no previous study provides the trends and patterns of men’s attitudes towards wife-beating in the country. The distinctive characteristic of this study is attributed to the inclusion of male samples taken from three rounds of nationally representative surveys. It also considers feminist theory, social learning theory, and ecological framework to identify several personal-, interpersonal-, household-, and community-level factors that are attributable to the development of tolerant attitudes of men towards wife-beating.

## Conclusion

A sizable percentage of men, more so those socio-economically marginalized, continue to justify wife-beating, albeit with considerable decline over the years. Nevertheless, the justification of wife-beating for her refusal to have sex is increasing. Men’s authoritarian attitudes towards women’s sexual autonomy and household decision-making, younger age, and a family history of IPV significantly elevate the risk of developing abusive attitudes towards wife-beating. The results indicate that the patriarchal mindset and existing gender norms are strongly linked to men's justification of violence against their wives. Additionally, structural factors such as economic status, educational attainment, alcohol use, and rural residence also significantly influence the attitude towards wife-beating. The findings suggest customized policies and programs enhancing gender egalitarian norms among young men, especially at the school level, providing more opportunities to pursue their higher education, alleviating poverty at the grassroots level by creating employment opportunities, raising awareness about domestic violence in rural settings through active social campaign, and promoting more equitable societal norms in everyday life would be helpful to develop more egalitarian gender norms and attitudes towards wife-beating.

### Supplementary Information

**Additional file 1: Table. 1A.** Classification table of regression analysis for justifying wife beating for at least one reason for all rounds of the survey. **Table. 2A.** Classification table of regression analysis for wife beating justified by unfaithfulness for all years. **Table. 3A.** Classification table of regression analysis for justifying wife beating for at least one reason, NFHS-3. **Table. 4A.** Classification table of regression analysis for justifying wife beating for unfaithfulness, NFHS-3. **Table. 5A.** Classification table of regression analysis for justifying wife beating for at least one reason, NFHS-4. **Table. 6A.** Classification table of regression analysis for justifying wife beating for unfaithfulness, NFHS-4. **Table. 7A.** Classification table of regression analysis for justifying wife beating for at least one reason, NFHS-5. **Table. 8A.** Classification table of regression analysis for justifying wife beating for unfaithfulness, NFHS-5.

## Data Availability

The datasets generated and/or analyzed during the current study are available in the Demographic and Health Surveys Repository (https://www.dhsprogram.com/data/new-user-registration.cfm). The repository allows data access through individual registration.

## Abbreviations

IPV: Intimate Partner Violence
NFHS: National Family Health Survey
CAPI: Computer Assisted Personal Interview
AOR: Adjusted Odds Ratio
CI: Confidence Interval
