# Supplementary material for: Men’s attitude towards wife-beating: understanding the pattern and trend in India
Source: BMC Public Health. 2024 Jan 31;24:331. doi: 10.1186/s12889-024-17782-w (PMC10829205; doi:10.1186/s12889-024-17782-w)
Supplement: Supplementary file 1 — Additional file 1: Table. 1A. Classification table of regression analysis for justifying wife beating for at least one reason for all rounds of the survey. Table. 2A. Classification table of regression analysis for wife beating justified by unfaithfulness for all years. Table. 3A. Classification table of regression analysis for justifying wife beating for at least one reason, NFHS-3. Table. 4A. Classification table of regression analysis for justifying wife beating for unfaithfulness, NFHS-3. Table. 5A. Classification table of regression analysis for justifying wife beating for at least one reason, NFHS-4. Table. 6A. Classification table of regression analysis for justifying wife beating for unfaithfulness, NFHS-4. Table. 7A. Classification table of regression analysis for justifying wife beating for at least one reason, NFHS-5. Table. 8A. Classification table of regression analysis for justifying wife beating for unfaithfulness, NFHS-5. [file 12889_2024_17782_MOESM1_ESM.docx]

**Additional File-1**

Table. 1A. Classification table of regression analysis for justifying wife beating for at least one reason for all rounds of the survey

| Logistic model for wife_beating justified at least one reason | | | |
| --- | --- | --- | --- |
|  | TRUE | | |
| Classified | D | ~D | Total |
|  |  |  |  |
| + | 59267 | 32807 | 92072 |
| - | 58695 | 125917 | 184612 |
|  |  |  |  |
| Total | 117962 | 158724 | 276686 |
| Classified + if predicted Pr(D) >= .5 |  |  |  |
| True D defined as wife_beating != 0 |  |  |  |
| Sensitivity |  | Pr( + D) | 50.24% |
| Specificity |  | Pr( -~D) | 79.38% |
| Positive predictive value |  | Pr( D +) | 64.44% |
| Negative predictive value |  | Pr(~D -) | 68.21% |
|  |  |  |  |
| False + rate for true ~D |  | Pr( +~D) | 20.62% |
| False - rate for true D |  | Pr( - D) | 49.76% |
| False + rate for classified | + | Pr(~D +) | 35.56% |
| False - rate for classified | - | Pr( D -) | 31.79% |
| Correctly classified |  |  | 66.95% |

Table. 2A. Classification table of regression analysis for wife beating justified by unfaithfulness for all years

| Logistic model for wife_beating justified by unfaithfulness | | | |
| --- | --- | --- | --- |
|  | TRUE | | |
| Classified | D | ~D | Total |
|  |  |  |  |
| + | 7612 | 6955 | 14567 |
| - | 61208 | 200911 | 262119 |
|  |  |  |  |
| Total | 68820 | 207866 | 276686 |
| Classified + if predicted Pr(D) >= .5 |  |  |  |
| True D defined as unfaith != 0 |  |  |  |
| Sensitivity |  | Pr( + D) | 11.06% |
| Specificity |  | Pr( -~D) | 96.70% |
| Positive predictive value |  | Pr( D +) | 52.60% |
| Negative predictive value |  | Pr(~D -) | 76.65% |
|  |  |  |  |
| False + rate for true ~D |  | Pr( +~D) | 3.30% |
| False - rate for true D |  | Pr( - D) | 88.94% |
| False + rate for classified | + | Pr(~D +) | 47.40% |
| False - rate for classified | - | Pr( D -) | 23.35% |
| Correctly classified |  |  | 75.39% |

Table. 3A. Classification table of regression analysis for justifying wife beating for at least one reason, NFHS-3

| Logistic model for wife-beating justified at least one reason | | | |
| --- | --- | --- | --- |
|  | TRUE |  |  |
| Classified | D | ~D | Total |
|  |  |  |  |
| + | 25095 | 11883 | 36978 |
| - | 10934 | 22692 | 33626 |
|  |  |  |  |
| Total | 36029 | 34575 | 70604 |
| Classified + if predicted Pr(D) >= .5 | |  |  |
| True D defined as wife_beating != 0 | |  |  |
| Sensitivity |  | Pr( + D) | 69.65% |
| Specificity |  | Pr( -~D) | 65.54% |
| Positive predictive value |  | Pr( D +) | 67.86% |
| Negative predictive value |  | Pr(~D -) | 67.39% |
|  |  |  |  |
| False + rate for true ~D |  | Pr( +~D) | 34.46% |
| False - rate for true D |  | Pr( - D) | 30.35% |
| False + rate for classified | + | Pr(~D +) | 32.14% |
| False - rate for classified | - | Pr( D -) | 32.61% |
| Correctly classified |  |  | 67.64% |

Table. 4A. Classification table of regression analysis for justifying wife beating for unfaithfulness, NFHS-3

| Logistic model for wife-beating justified by unfaithfulness | | | |
| --- | --- | --- | --- |
|  | TRUE |  |  |
| Classified | D | ~D | Total |
|  |  |  |  |
| + | 3685 | 3297 | 6982 |
| - | 16120 | 47502 | 63622 |
|  |  |  |  |
| Total | 19805 | 50799 | 70604 |
| Classified + if predicted Pr(D) >= .5 |  |  |  |
| True D defined as unfaith != 0 |  |  |  |
| Sensitivity |  | Pr( + D) | 18.61% |
| Specificity |  | Pr( -~D) | 93.68% |
| Positive predictive value |  | Pr( D +) | 53.51% |
| Negative predictive value |  | Pr(~D -) | 74.66% |
|  |  |  |  |
| False + rate for true ~D |  | Pr( +~D) | 6.32% |
| False - rate for true D |  | Pr( - D) | 81.39% |
| False + rate for classified | + | Pr(~D +) | 46.49% |
| False - rate for classified | - | Pr( D -) | 25.34% |
| Correctly classified |  |  | 72.60% |

Table. 5A. Classification table of regression analysis for justifying wife beating for at least one reason, NFHS-4

| Logistic model for wife_beating justified at least one reason | | | |
| --- | --- | --- | --- |
|  | TRUE |  |  |
| Classified | D | ~D | Total |
|  |  |  |  |
| + | 18523 | 10947 | 29470 |
| - | 24687 | 53650 | 78337 |
|  |  |  |  |
| Total | 43210 | 64597 | 107807 |
| Classified + if predicted Pr(D) >= .5 |  |  |  |
| True D defined as wife_beating != 0 |  |  |  |
| Sensitivity |  | Pr( + D) | 42.87% |
| Specificity |  | Pr( -~D) | 83.05% |
| Positive predictive value |  | Pr( D +) | 62.85% |
| Negative predictive value |  | Pr(~D -) | 68.49% |
|  |  |  |  |
| False + rate for true ~D |  | Pr( +~D) | 16.95% |
| False - rate for true D |  | Pr( - D) | 57.13% |
| False + rate for classified | + | Pr(~D +) | 37.15% |
| False - rate for classified | - | Pr( D -) | 31.51% |
| Correctly classified |  |  | 66.95% |

Table. 6A. Classification table of regression analysis for justifying wife beating for unfaithfulness, NFHS-4

| Logistic model for wife_beating justified by unfaithfulness | | | |
| --- | --- | --- | --- |
|  | TRUE |  |  |
| Classified | D | ~D | Total |
|  |  |  |  |
| + | 2784 | 2437 | 5221 |
| - | 23229 | 79357 | 102586 |
|  |  |  |  |
| Total | 26013 | 81794 | 107807 |
| Classified + if predicted Pr(D) >= .5 |  |  |  |
| True D defined as unfaith != 0 |  |  |  |
| Sensitivity |  | Pr( + D) | 10.70% |
| Specificity |  | Pr( -~D) | 97.02% |
| Positive predictive value |  | Pr( D +) | 53.32% |
| Negative predictive value |  | Pr(~D -) | 77.36% |
|  |  |  |  |
| False + rate for true ~D |  | Pr( +~D) | 2.98% |
| False - rate for true D |  | Pr( - D) | 89.30% |
| False + rate for classified | + | Pr(~D +) | 46.68% |
| False - rate for classified | - | Pr( D -) | 22.64% |
| Correctly classified |  |  | 76.19% |

Table. 7A. Classification table of regression analysis for justifying wife beating for at least one reason, NFHS-5

| Logistic model for wife_beating justified at least one reason | | | |
| --- | --- | --- | --- |
|  | TRUE | | |
| Classified | D | ~D | Total |
|  |  |  |  |
| + | 17775 | 9181 | 26956 |
| - | 20948 | 50371 | 71319 |
|  |  |  |  |
| Total | 38723 | 59552 | 98275 |
| Classified + if predicted Pr(D) >= .5 |  |  |  |
| True D defined as wife_beating != 0 |  |  |  |
| Sensitivity |  | Pr( + D) | 45.90% |
| Specificity |  | Pr( -~D) | 84.58% |
| Positive predictive value |  | Pr( D +) | 65.94% |
| Negative predictive value |  | Pr(~D -) | 70.63% |
|  |  |  |  |
| False + rate for true ~D |  | Pr( +~D) | 15.42% |
| False - rate for true D |  | Pr( - D) | 54.10% |
| False + rate for classified | + | Pr(~D +) | 34.06% |
| False - rate for classified | - | Pr( D -) | 29.37% |
| Correctly classified |  |  | 69.34% |

Table. 8A. Classification table of regression analysis for justifying wife beating for unfaithfulness, NFHS-5

| Logistic model for wife_beating justified by unfaithfulness | | | |
| --- | --- | --- | --- |
|  | TRUE | | |
| Classified | D | ~D | Total |
|  |  |  |  |
| + | 3378 | 2779 | 6157 |
| - | 19624 | 72494 | 92118 |
|  |  |  |  |
| Total | 23002 | 75273 | 98275 |
| Classified + if predicted Pr(D) >= .5 |  |  |  |
| True D defined as unfaith != 0 |  |  |  |
| Sensitivity |  | Pr( + D) | 14.69% |
| Specificity |  | Pr( -~D) | 96.31% |
| Positive predictive value |  | Pr( D +) | 54.86% |
| Negative predictive value |  | Pr(~D -) | 78.70% |
|  |  |  |  |
| False + rate for true ~D |  | Pr( +~D) | 3.69% |
| False - rate for true D |  | Pr( - D) | 85.31% |
| False + rate for classified | + | Pr(~D +) | 45.14% |
| False - rate for classified | - | Pr( D -) | 21.30% |
| Correctly classified |  |  | 77.20% |
